# Progress toward UNAIDS 90-90-90 targets: A respondent-driven survey among female sex workers in Kampala, Uganda

**DOI:** 10.1371/journal.pone.0201352

**Published:** 2018-09-19

**Authors:** Reena H. Doshi, Enos Sande, Moses Ogwal, Herbert Kiyingi, Anne McIntyre, Joy Kusiima, Geofrey Musinguzi, David Serwadda, Wolfgang Hladik

**Affiliations:** 1 Epidemic Intelligence Service, Centers for Disease Control and Prevention, Atlanta, GA, United States of America; 2 Division of Global HIV and TB, Center for Global Health, Centers for Disease Control and Prevention, Atlanta, GA, United States of America; 3 Division of Global HIV and TB, Centers for Disease Control and Prevention, Entebbe, Uganda; 4 Makerere University School of Public Health, Kampala, Uganda; Simon Fraser University, CANADA

## Abstract

**Background:**

We investigated progress towards UNAIDS 90-90-90 targets among female sex workers in Kampala, Uganda, who bear a disproportionate burden of HIV.

**Methods:**

Between April and December 2012, 1,487 female sex workers, defined as women, 15–49 years, residing in greater Kampala, and selling sex for money in the last 6 months, were recruited using respondent-driven sampling. Venous blood was collected for HIV and viral load testing [viral load suppression (VLS) defined as <1,000 copies/mL]. We collected data using audio computer-assisted self-interviews and calculated weighted population-level estimates.

**Results:**

The median age was 27 years (interquartile range: 23 to 32). HIV seroprevalence was 31.4% (95% confidence interval [CI]: 29.0, 33.7%). Among all female sex workers who tested HIV-positive in the survey (population-level targets), 45.5% (95% CI: 40.1, 51.0) had knowledge of their serostatus (population-level target: 90%), 37.8% (95% CI: 32.2, 42.8) self-reported to be on ART (population-level target: 81%), and 35.2% (95% CI: 20.7, 30.4) were virally suppressed (population-level target: 73%).

**Conclusions:**

HIV prevalence among Kampala female sex workers is high, whereas serostatus knowledge and VLS are far below UNAIDS targets. Kampala female sex workers are in need of intensified and targeted HIV prevention and control efforts.

## Introduction

In 2014, the Joint United Nations Programme on HIV/AIDS (UNAIDS) launched three treatment targets to achieve epidemic control by 2020; that is, 90% of all people living with HIV will know their HIV status, 90% of those diagnosed with HIV infection will receive antiretroviral therapy (ART), and 90% of all people receiving ART will have viral suppression (90-90-90 targets)[1].

Key populations such as female sex workers are considered a priority population in efforts to achieve 90-90-90 targets[1]. Female sex workers bear a disproportionately large burden of global HIV infections and are estimated to be 13.5 times more likely to become infected than the general population of women in low and middle income countries[2]. Multiple and concurrent sexual partners, inconsistent condom use, and a high prevalence of sexually transmitted infections (STI) alongside structural determinants expose female sex workers and, subsequently, their clients and partners to a greater risk of HIV acquisition[2]. In sub-Saharan Africa, an estimated 18% of the total HIV burden among women 15 years or older has been attributed to female sex work [3] and the estimated HIV prevalence among female sex workers is 36.9%[2].

Uganda has a mature, generalized HIV epidemic; however, sex work is likely an important driver of sexual transmission[4]. According to the Uganda AIDS Commission, sex work accounted for an estimated 11% of new infections in 2009[4]. A 2009 study found an HIV prevalence of 33% among Kampala female sex workers [5] compared to 9.5% among the general population of Kampala females of reproductive age[6]. Despite that, access to HIV prevention and treatment services for female sex workers is limited[2, 5]. The high prevalence of HIV in Uganda suggests that there are significant barriers for female sex workers to obtain essential health services[7, 8]. In Uganda, sex work remains criminalized, increasing stigma and marginalization[9]. Factors such as poverty, discrimination, gender inequality, severe physical violence, and criminalization of sex work increase female sex worker’s risk for infection and deter these women from learning their HIV status or accessing prevention and treatment services[7, 10].

Scale-up of prevention and treatment programs could reduce HIV transmission among female sex workers, however, the effectiveness of these interventions are dependent on the extent to which Ugandan female sex workers engage in prevention and treatment services. Being tested and knowing one’s HIV status is associated with a reduction in HIV risk behaviors, prevents onward transmission, and can lead to mobilization of support networks [11], increasing the ability to make informed decisions and, in turn, utilize necessary health services[12]. There is a dearth of population-level data examining the burden of HIV among female sex workers and the uptake of HIV services (from testing and diagnosis to virological suppression), mainly due to challenges in representative population-based sampling[13]. To address this gap in knowledge, we utilized data from the 2012 Crane Survey, a key population focused survey in Kampala, Uganda. We report here on the estimated prevalence of HIV among female sex workers, the population-level progress toward 90-90-90 targets, and factors associated with serostatus knowledge, ART use, and viral load suppression (VLS).

## Methods

### Study design

Female sex workers were recruited using respondent driven sampling (RDS), a modified form of chain-referral sampling with a mathematical system for weighting, suitable for hard-to-sample populations[14, 15]. Recruitment occurred from April to December 2012. Women were eligible if they were 15 years or older at the time of recruitment, living in greater Kampala, and sold sex to men in the preceding 6 months. Survey staff identified and enrolled four female sex workers as “seeds” (initial participants) to initiate recruitment of other female sex workers in their social networks through the use of coupons. Seeds were well-connected within their networks, well regarded by their peers, sympathetic to the survey’s goals and diverse with regard to education, socioeconomic status, age, and place of residence. These “waves” of recruitment were repeated until the desired sample size was achieved (maximum number of waves achieved: 25). All variables of interest reached convergence. Participating women were provided ($4.00 USD) for their time and travel to the interview site, in addition to an incentive for each successful peer recruitment ($1.25 USD per recruit). Initially, each participant was provided three coupons; this was later reduced to two coupons, then to one, and finally to zero, to bring sampling to a controlled end.

### Data collection

Eligible and consenting women were interviewed using audio computer assisted self-interview (ACASI) in either Luganda or English. The interview’s key domains included demographics, lifetime sexual characteristics, sexual behaviors in the last three months, sexual violence, and HIV testing and treatment history. Other data measures included alcohol use and drug use (including injection drug use).

### HIV testing

All HIV testing was voluntary. Ugandan ministry of guidelines for HIV counseling, testing and referral services were followed[16]. Pre-test counseling included an explanation of HIV infection and transmission, the meaning of HIV test results, risks associated with sexual behaviors, as well as means for HIV prevention. Post-test counseling messages were tailored to recruits’ HIV result and risk profiles and included goals, means, and strategies for behavioral risk reduction, maintenance of risk reduction, and explanation of risk reduction methods (e.g., condom use, drug use). Counseling also included an assessment of psychosocial needs, a discussion of living with HIV-infection, treatment and care, and issues related to discrimination. All HIV-positive FSW were provided a referral and study staff offered to accompany the individual.

### Laboratory procedures

At their initial visit, female sex workers provided a venous blood sample for HIV and syphilis testing and, if HIV-positive, for CD4+ T cell count and viral load. HIV serologic testing was conducted at the Uganda Virus Research Institute (UVRI) laboratory in Entebbe using Vironostika® Uniform II plus O2, 3^rd^ generation (bioMeriéux, Marcy l’Etoile, France) and Murex HIV Ab, 3^rd^ generation (Abbott Laboratories, Abbott Park, Illinois, U.S.A.) in parallel. STAT-PAK (Inverness Medical, Princeton, New Jersey, U.S.A.) was used as a tiebreaker for discordant results. Respondents with concordent reactive results or STAT-PAK reactive results were classified as HIV seropositive. HIV seropositive specimens were further tested for viral load using COBAS TaqMan® assay. An undetectable viral load was defined as <50 copies/ml and viral load suppression was defined as <1000 copies/ml, chosen based on World Health Organization guidelines[17]. Plasma was also tested for *Treponema pallidum* (TP) infection, using the anti-syphilis IgG ELISA (Biotec Laboratories, Suffolk, UK) and, if reactive, the rapid plasma reagin (RPR) Syfacard-R Test (Murex Biotech, Dartford, UK). Respondents with RPR-reactive test results were classified as having active TP infection.

### Statistical methods

In total, 1,497 female sex workers were included in the analysis, including 2 of the 4 seeds. Two seeds were unproductive and did not recruit other female sex workers. RDS Analyst (RDS-A) v5.7 was used to generate unweighted and weighted descriptive statistics[18]. We utilized Gile’s sequential sampling estimator[19], with 100,000 resamples for bootstrapping to generate weighted population estimates and 95% confidence intervals (CI) for demographic and behavioral characteristics.

We defined serostatus knowledge as an HIV seropositive female sex workers who reported a previous positive test result. Female sex workers were classified as on ART if she self-reported ART use among those who reported a previous positive test result. VLS was defined as <1,000 copies/ml among those female sex workers self-reporting ART use.

Population-level estimates of each unconditional 90 target were calculated using all HIV seropositive FSW as the denominator, i.e., the proportion of all HIV seropositive female sex workers who reported a previous positive test, reported ART use, or were virally suppressed.

Weighted logistic regression was used to examine bivariate associations between serostatus knowledge, ART use, or viral suppression and demographic characteristics, sexual behaviors, substance use, drug use, HIV knowledge, and STI history. All regression analyses were conducted using SAS 9.4 (SAS Institute Inc., Cary, NC).

### Sensitivity analyses

We conducted a sensitivity analysis for population-level estimates of 90-90-90 targets by assuming that any female sex workers with viral suppression was taking ART at the time of the study, and further that all female sex workers on ART knew their serostatus. We also varied the definition of viral load suppression. We calculated population level estimates for 90-90-90 targets with viral load suppression defined as <50 copies/ml and <200 copies/ml.

The study protocol and consent procedures were approved by the human subjects protection boards at Makerere University School of Public Health, the Uganda National Council of Science and Technology (UNCST), and Centers for Disease Control and Prevention (CDC). Informed consent was obtained verbally and separately for interview, blood draw and testing, as well as storage of specimens for future testing. No personal identifiers were collected; biomarker results were returned to recruits at their scheduled return visit, three weeks following the initial visit. Recruits testing positive for syphilis were offered treatment; those testing HIV-positive were referred to care and ART per national guidelines. Female sex workers between the ages of 15–17 years were treated as emancipated minors and provided informed consent themselves.

## Results

### Sampling and demographics

Of 4,018 coupons issued, 1,915 (47.7%) were redeemed. Of the 1,915 redeemed, 1,501 (78.4%) women were eligible to participate and 1,497 completed the visit and were included in the analyses. Women were ineligible if they had not sold sex in the 6 months prior and were under the age of 15. Unweighted demographic characteristics and weighted population estimates are reported in Table 1. The median age of participating female sex workers was 27 years (interquartile range [IQR]: 23 to 32), 34.3% [95% Confidence Interval (C1): 30.7, 37.9] were Catholic and 37.6% (95% CI: 34.7, 40.5) had no years of schooling. About half (49.5%, 95% CI: 46.5, 52.4%) were never married; and 54.7% (95% CI: 51.5, 57.9) started sex work at 25 years of age or older. Sex work was the main source of income for 94.4% (95% CI: 93.0, 95.9), and the median number of years engaged in sex work was 2 (IQR: 1–4). An estimated 54.6% of female sex workers, 95% CI: 51.3, 57.8) had three or more children and 8.6% (95% CI: 6.9, 10.2) were currently pregnant. Approximately one quarter [26.0% (95% CI: 23.3, 28.6%)] of female sex workers reported meeting clients on the street and 28.3% (95% CI: 25.4, 31.2%) met clients at bars, clubs, or restaurants.

**Table 1:** Demographic characteristics for female sex workers, crude and weighted results, Crane Survey, Kampala, Uganda, 2012 (n = 1,497).

Characteristic	n	%	Weighted % (95% CI)^1^
Age, in years (median, IQR^1^)	28 (23–32)		27 (23–32)
15–24	480	32.1	32.7 (29.4–35.9)
25–34	759	50.7	50.0 (26.9–53.1)
35–49	258	17.2	17.3 (14.9–19.7)
Religion			
Protestant	433	29.2	29.4 (25.9–32.9)
Catholic	543	36.6	34.3 (30.7–37.9)
Muslim	412	27.8	29.1 (25.7–32.5)
Other	83	5.6	6.0 (4.1–7.8)
None	13	0.9	1.2 (0.4–2.0)
Schooling, in years (median, IQR)	6 (0–10)		6 (0–9)
None	539	36.2	37.6 (34.7–40.5)
1–7	458	30.8	30.6 (27.9–33.4)
8–13	357	24	23.3 (20.6–25.9)
≥14	133	8.9	8.5 (6.7–10.2)
Current marital status			
Never married	747	50.2	49.5 (46.5–52.4)
Married	91	6.1	5.9 (4.6–7.3)
Divorced	272	18.3	19.0 (16.6–21.4)
Separated	302	20.3	20.2 (17.8–22.7)
Widow	75	5	5.3 (3.9–6.7)
Age at starting sex work, in years			
<25	692	46.6	45.3 (42.1–48.5)
≧25	792	53.4	54.7 (51.5–57.9)
Sex work as main source of income			
Yes	1401	94.2	94.4 (93.0–95.9)
No	86	5.8	5.6 (4.1–7.0)
Years as a sex worker (median, IQR)	2 (1–5)		2 (1–4)
<1	302	20.3	22.2 (19.5–24.9)
1–2	483	32.5	35.6 (32.6–38.7)
3–5	439	29.5	26.5 (24.0–29.1)
≥ 6	263	17.7	15.6 (13.5–17.8)
Number of children (median, IQR)	3 (2–4)		3 (2–4)
0	37	2.8	3.0 (1.7–4.3)
1	236	18.1	17.4 (15.0–19.8)
2	317	24.3	25.0 (22.3–27.7)
≥3	717	54.9	54.6 (51.3–57.8)
Currently pregnant			
No	1195	91.4	91.4 (89.8–93.1)
Yes	112	8.6	8.6 (6.9–10.2)
Location of client pick-up			
Street	407	27.4	26.0 (23.3–28.6)
Phone/internet	205	13.8	13.0 (11.0–15.0)
Hotel	227	15.3	16.2 (14.0–18.4)
Club, bar, restaurant	414	27.8	28.3 (25.3–31.2)
Private place	132	8.9	9.8 (7.9–11.7)
Brothel	102	6.9	6.8 (5.2–8.4)

^1^IQR: interquartile range.

CI: confidence interval.

### HIV prevalence and testing characteristics

The estimated HIV prevalence was 31.4% (95% CI: 29.0, 33.7%) (Table 2). Among all female sex workers, 71.9% (95% CI: 69.0, 74.9) of female sex workers indicated they had been previously tested for HIV at some time, whereas 67.6% indicated they had been tested within the past 12 months. Of those ever tested, 13.6% (95% CI: 11.0, 16.3) reported a positive HIV test result. Among all female sex workers self-reporting a positive HIV test result, only 3.7% (95% CI: 2.6, 4.8) of female sex workers perceived themselves to be HIV-positive at the time of the interview.

**Table 2:** HIV testing and treatment characteristics for female sex workers by HIV serostatus, Crane Survey, Kampala, Uganda, 2012 (n = 1,497).

	All Participants			HIV-Positive (n = 485)			HIV-Negative (n = 1,007)		
	n	%	Weighted % (95% CI)	n	%	Weighted % (95% CI)	n	%	Weighted % (95% CI)
**HIV status**									
** Negative**	1007	67.5	68.7 (66.3–71.0)	-	-	-	-	-	-
** Positive**	485	32.5	31.4 (29.0–33.7)						
**Active syphilis infection**									
** Negative**	1395	93.6	93.8 (87.1–100.0)	445	91.8	91.7 (88.9–94.5)	950	94.5	94.7 (92.1–97.3)
** Positive**	95	6.4	6.2 (0–12.8)	40	8.2	8.3 (5.5–11.1)	55	5.5	5.4 (2.7–7.9)
**Ever tested for HIV**									
** No**	418	28.1	28.1 (25.1–31.0)	219	45.4	54.9 (49.2–60.5)	197	19.7	20.2 (17.1–23.4)
** Yes**	1069	71.9	71.9 (69.0–74.9)	263	54.6	45.1 (39.5–50.8)	803	80.3	79.8 (76.7–82.9)
**HIV test in the last 12 months**									
** No**	335	31.4	32.4 (29.1–35.8)	106	40.3	38.9 (31.8–46.2)	228	28.5	30.3 (26.5–34.2)
** Yes**	731	68.6	67.6 (64.2–70.9)	157	59.7	61.1 (53.8–68.2)	572	71.5	69.7 (65.8–73.5)
**Self-reported last test result^1^**									
** Positive**	145	13.6	13.6 (11.0–16.3)	123	46.8	48.7 (41.5–56.0)	22	2.7	2.7 (1.2–4.1)
** Negative**	854	79.9	79.5 (76.5–82.4)	102	38.8	36.8 (30.1–43.4)	749	93.3	92.8 (90.6–95.0)
** Unknown**	70	6.5	6.9 (5.2–8.6)	38	14.4	14.5 (9.5–19.4)	32	4	4.5 (2.9–6.2)
**Self-perceived HIV status^2^**									
** Positive**	52	3.9	3.7 (2.6–4.8)	28	7.8	7.8 (4.8–10.8)	23	2.4	2.2 (1.3–3.2)
** Negative**	318	23.7	23.5 (20.8–26.2)	45	12.5	12.9 (8.5–17.3)	272	27.8	27.2 (23.9–30.6)
** Don’t Know**	972	72.4	72.7 (70.0–75.5)	286	79.7	79.3 (74.4–84.3)	683	69.8	70.5 (67.6–73.9)
**Self-reported ART use^3^**									
** Yes**	-	-	-	86	69.9	67.7 (56.1–78.8)	-	-	-
** No**				37	30.1	32.3 (21.2–43.9)			
**Viral load suppression^4^^,^^5^**									
** Suppressed**	127	8.5	8.1 (6.5–9.7)	28	53.8	51.6 (34.8–68.5)	-	-	-
** Unsuppressed**	218	14.6	14.7 (12.7–16.8)	24	46.2	48.4 (31.5–65.2)			

^1^Denominator is those who indicated they had been previously tested for HIV

^2^Demoninator is all FSW

^3^Among those who reported a previous positive test result (n = 123)

^4^Among those who self-reported ART use (n = 86), viral load suppression defined as <1000 copies/ml, 34 FSW were excluded due to missing viral load data

^5^Note that only HIV positive FSW were tested for viral load and 1152 (77%) are not included

Among HIV seropositive female sex workers, 48.7% (95% CI: 41.5, 56.0%) reported a previous positive HIV test result, whereas only 7.8% (95% CI: 4.8, 10.8%) perceived themselves to be HIV positive. Among seropositive female sex workers who reported a previous positive HIV test result (n = 123), 67.7% (95% CI: 56.1, 78.8%) indicated they were taking ART. An estimated 51.6% (95%CI: 34.8, 68.5%) of female sex workers were virally suppressed among women who self-reported ART use. The prevalence of unsuppressed viremia (i.e., the prevalence of HIV-positivity with a VL ≥ 1,000 copies among all female sex workers) was estimated at 14.7% (95% CI: 12.7, 16.8%)

### Population-level 90-90-90 estimates

**Fig 1:**
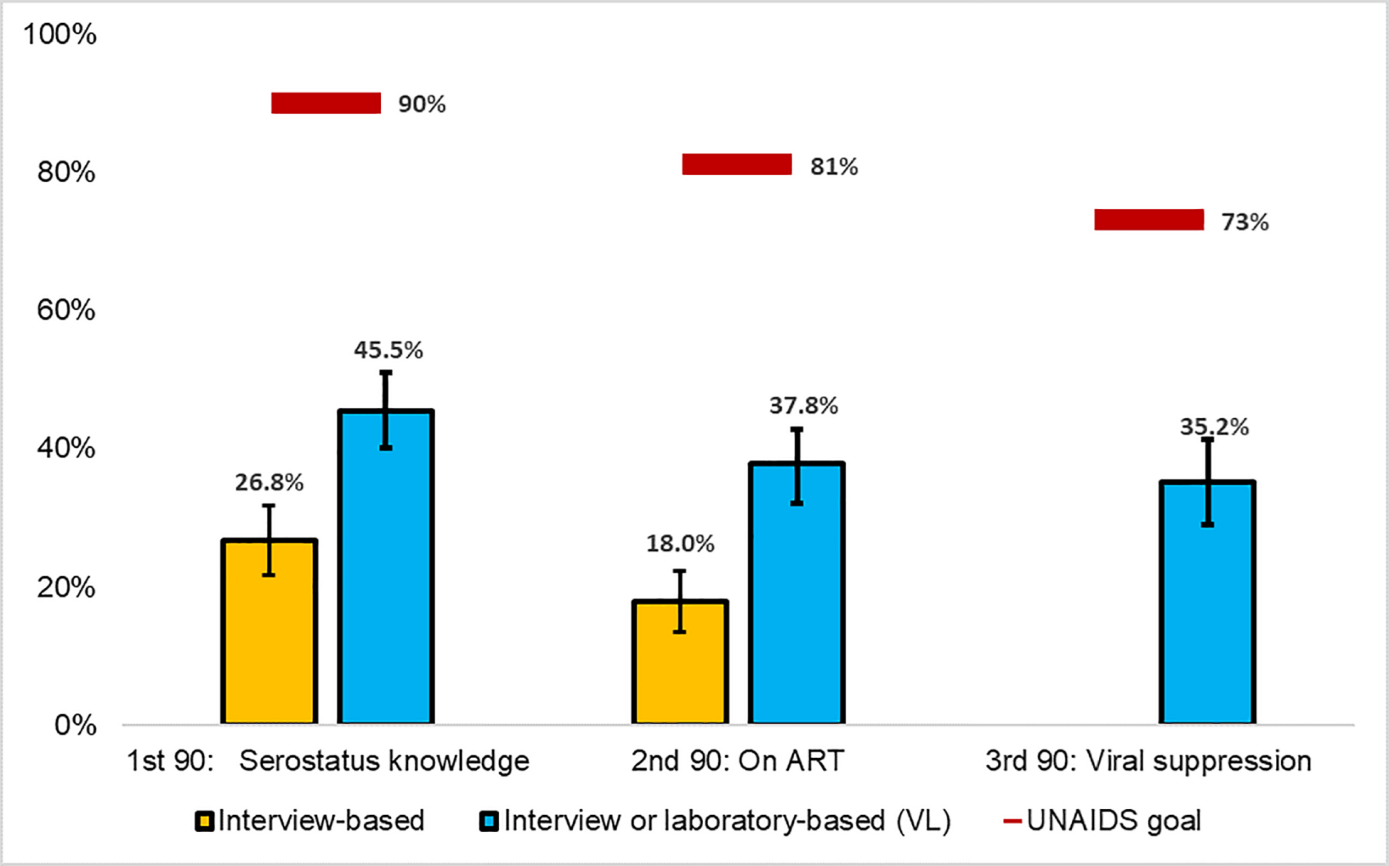
FSW serosurvey results compared with population-level UNAIDS 90-90-90 targets, Crane Survey, Kampala, Uganda, 2012. ^1^Serostatus knowledge (yellow) includes all HIV positive women who self-reported a previous positive test result; blue column also includes FSW with viral suppression. ^2^On ART (yellow) includes all HIV positive women who self-reported taking ARTs; blue column also includes FSW with viral suppression. ^3^Viral suppression was laboratory-based and defined as <1000 copies/ml. ^4^Denominator for viral suppression was n = 341 due to missing data; the denominator for all other characteristics was n = 485. ^5^Error bars represent 95% confidence intervals.

Among seropositive female sex workers, serostatus knowledge was 26.8%, 18% reported being on ART, and 35.2% were virally suppressed (Fig 1). Under the assumption that virally suppressed (<1,000 copies/ml) female sex workers were on ART and thus knew their serostatus, knowledge increased to 45.5% and the proportion of those on ART increased to 37.8%.

**Fig 2:**
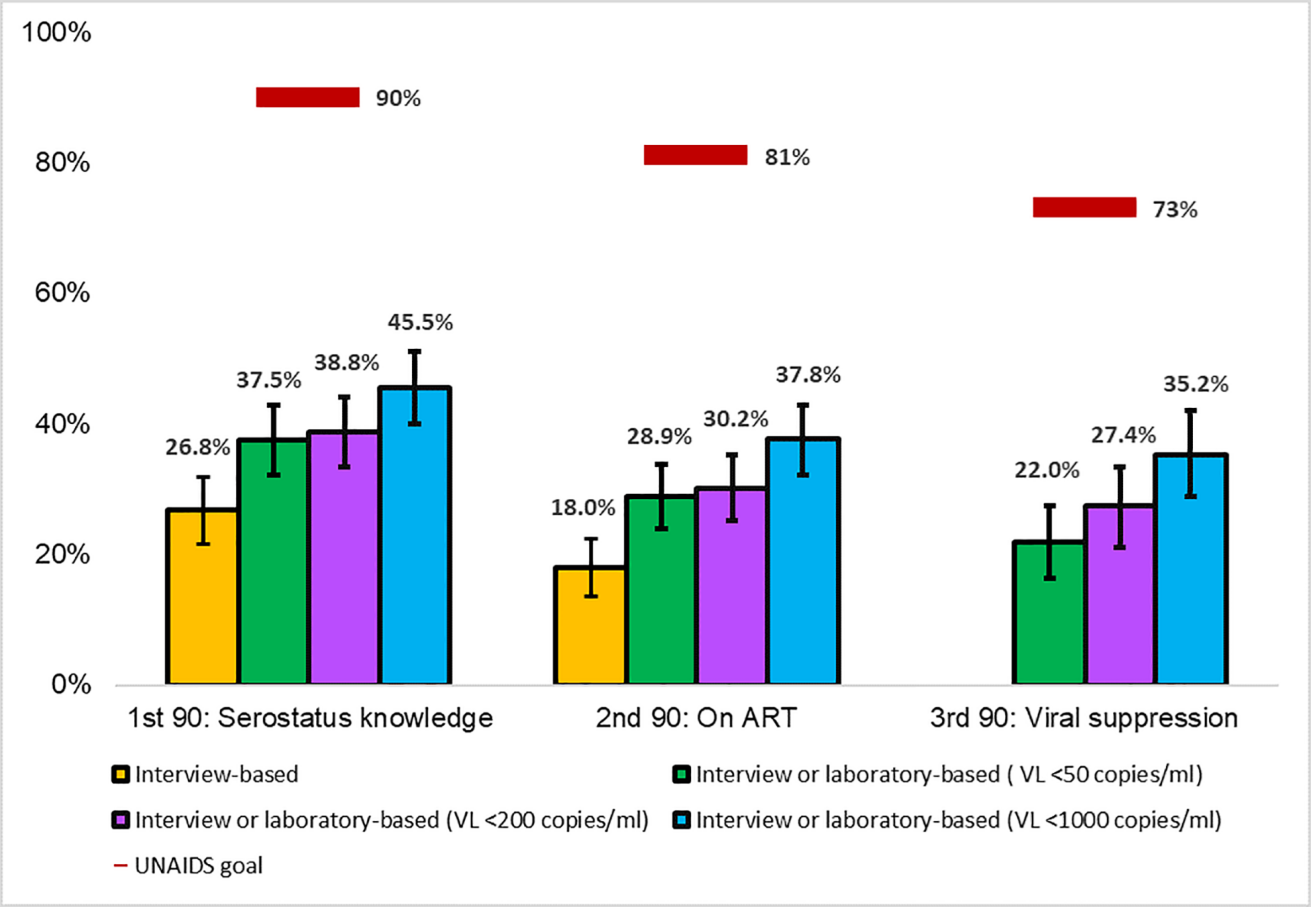
FSW serosurvey results compared with population-level UNAIDS 90-90-90 targets by selected viral load suppression thresholds, Crane Survey, Kampala, Uganda, 2012. ^1^Serostatus knowledge (yellow) includes all HIV positive women who self-reported a previous positive test result; blue column also includes FSW with viral suppression. ^2^On ART (yellow) includes all HIV positive women who self-reported taking ARTs; blue, green, purple columns also includes FSW with viral suppression by definition. ^3^Denominator for viral suppression was n = 341 due to missing data; the denominator for all other characteristics was n = 485. ^4^Error bars represent 95% confidence intervals.

When viral load suppression was defined as <200 copies/ml, serostatus knowledge, ART use, and viral load suppression decreased to 38.8%, 30.2%, and 27.4%, respectively (Fig 2). When viral load suppression was defined as <50 copies/ml, serostatus knowledge, ART use, and viral load suppression declined to 37.5% 28.9%, and 22.0%, respectively.

### Correlates of being outside the 90-90-90 targets

Female sex workers who started sex work at the age of 25 or older had 0.57 (95% CI: 0.33, 0.99) times the odds of not knowing their serostatus (Table 3) and 0.47 (95% CI: 0.20, 1.08) times the odds of not being on ART compared to female sex workers who started sex work under the age of 25. Compared to protestant female sex workers, catholic female sex workers had 2.26 (95% CI: 1.14, 4.48) times the odds of unsuppressed viremia. Female sex workers who had been working 3 to 5 years had 0.53 (95% CI: 0.29, 0.96) times the odds of having unsuppressed viremia compared to those who had been working for two years or less. Finally, women who met clients in a hotel/bar/restaurant/brothel/private place had 0.59 (95% CI: 0.33, 1.07) times the odds of having unsuppressed viremia compared to who women who worked on the street.

**Table 3:** Correlates of being outside the 90-90-90 targets and sex worker characteristics among HIV positive female sex workers, Crane Survey, Kampala, Uganda, 2012.

	Serostatus Knowledge (n = 321)^1^^,^^2^					ART Use (n = 216)^4^					Viral Load Suppression (n = 341)^5^				
	Serostatus unknown		Serostatus known			Not on ART		On ART			No VLS		VLS		
	n	%	n	%	Weighted OR (95% CI)	n	%	n	%	Weighted OR (95% CI)	n	%	n	%	Weighted OR (95% CI)
**Age, in years**															
**15–24**	36	34.3	48	22.2	Ref	11	35.5	37	20.3	Ref	68	31.3	20	16.1	Ref
**25–49**	69	65.7	168	77.8	0.54 (0.37–0.80)	20	64.5	145	79.7	0.61 (0.24–1.52)	149	68.7	104	83.9	0.58 (0.30–1.15)
**Religion**															
**Protestant**	32	30.5	68	31.8	Ref	10	29.4	58	32.2	Ref	56	26.2	42	34.4	Ref
**Catholic**	46	43.8	85	39.7	1.23 (0.63–2.37)	15	44.1	70	38.9	1.30 (0.48–3.52)	95	44.4	38	31.1	2.26 (1.14–4.48)
**Other**	27	25.7	61	28.5	0.71 (0.34–1.50)	9	26.5	52	28.9	0.74 (0.24–2.31)	63	29.4	42	34.4	0.87 (0.44–1.72)
**Years as a sex worker**															
**0–2**	59	56.2	94	43.7	Ref	17	50	77	42.5	Ref	110	51.2	48	39	Ref
**3–5**	28	26.7	67	31.2	0.65 (0.34–1.22)	10	29.4	57	31.5	1.02 (0.38–2.71)	66	30.7	44	35.8	0.53 (0.29–0.96)
**≥6**	18	17.1	54	25.1	0.66 (0.31–1.41)	7	20.6	47	26	0.88 (0.29–2.69)	39	18.1	31	25.2	0.56 (0.29–1.08)
**Schooling, in years**															
**None**	37	35.2	80	37.2	Ref	12	35.3	68	37.6	Ref	90	41.9	44	35.8	Ref
**1–7**	33	31.4	75	34.9	1.12 (0.58–2.15)	11	32.4	64	35.4	1.12 (0.40–3.08)	61	28.4	45	36.6	0.38 (0.21–0.69)
**≥8**	35	33.3	60	27.9	1.36 (0.69–2.68)	11	32.4	49	27.1	0.76 (0.26–2.17)	64	29.8	34	27.6	0.63 (0.32–1.26)
**Marital status**															
**Never married**	60	57.1	99	46	Ref	15	44.1	84	46.4	Ref	108	50.2	57	46.3	Ref
**Ever married**	45	42.9	116	54	0.90 (0.50–1.64)	19	55.9	97	53.6	0.66 (0.29–1.51)	107	49.8	66	53.7	1.09 (0.64–1.85)
**Number of children**															
**0–2**	43	47.3	83	42.6	Ref	15	46.9	68	41.7	Ref	94	49	39	35.1	Ref
**≥3**	48	52.7	112	57.4	0.90 (0.50–1.64)	17	53.1	95	58.3	1.11 (0.46–2.65)	98	51	72	64.9	0.78 (0.44–1.39)
**Work location**															
**Street**	25	23.8	63	29.3	Ref	12	35.3	51	28.2	Ref	76	35.2	32	26	Ref
**Phone/internet**	20	19	38	17.7	0.84 (0.37–1.91)	7	20.6	31	17.1	0.70 (0.21–2.31)	25	11.6	19	15.5	0.57 (0.24–1.37)
**Hotel, bar, restaurant, brothel or private place**	60	57.1	114	53	0.98 (0.52–1.84)	15	44.1	99	54.7	1.22 (0.47–3.13)	115	53.2	72	58.5	0.59 (0.33–1.07)
**Alcohol use^3^**															
**Infrequent use**	56	58.3	105	53.8	Ref	17	51.5	91	56.2	Ref	106	54.6	56	51.4	Ref
**Frequent use**	40	41.7	90	46.2	0.82 (0.46–1.47)	16	48.5	71	43.8	1.24 (0.52–2.98)	88	45.4	53	48.6	0.81 (0.46–1.43)
**Ever injected drugs**															
**No**	46	76.7	74	77.1	Ref	12	80	62	76.5	Ref	65	63.7	40	76.9	Ref
**Yes**	14	23.3	22	22.9	1.08 (0.42–2.75)	3	20	19	23.5	1.91 (0.38–9.54)	37	36.3	12	23.1	2.05 (0.81–5.20)
**Forced into sex work**															
**No**	39	37.1	71	33	Ref	23	67.6	121	66.9	Ref	77	35.8	39	31.7	Ref
**Yes**	66	62.9	144	67	0.80 (0.55–1.42)	11	32.4	60	33.1	0.98 (0.40–2.43)	138	64.2	84	68.3	0.67 (0.38–1.20)
**Age at starting sex work, in years**															
**<25**	50	47.6	81	37.7	Ref	19	55.9	62	34.3	Ref	103	47.9	35	28.5	Ref
**≥25**	55	52.4	134	62.3	0.57 (0.33–0.99)	15	44.1	119	65.7	0.47 (0.20–1.08)	112	52.1	88	71.5	0.55 (0.31–0.97)
**Active syphilis infection**															
**No**	94	89.5	194	90.2	Ref	28	82.4	167	91.8	Ref	196	90.3	118	94.4	Ref
**Yes**	11	10.5	21	9.8	0.85 (0.36–2.03)	6	17.6	15	8.2	2.06 (0.65–6.54)	21	9.7	7	5.6	1.50 (0.55–4.12)

^1^Analysis excludes FSW who did not indicate they had been previously tested, did not indicate a previous positive test results, and were not virally suppressed.

^2^Serostatus known (refererent) includes 35 FSW with virally suppression, defined as <1000 copies/ml

^3^Infrequent use defined as either never drinking alcohol or drinking a couple of times a month, frequent use defined as drinking two or more times per week

^4^216 includes 96 FSW who were virally suppressed (<1000 copies/ml); 269 of the 485 HIV positive FSW did not have data on ART use and were not virally suppressed

^5^Viral load suppression (VLS) is defined as <1000 copies

## Discussion

HIV prevalence among Kampala female sex workers (31.4%) is more than three times the HIV prevalence of the general female population of reproductive age in Kampala (9.5%)[6]. The high prevalence of unsuppressed viremia (14.7%) among seropositive female sex workers is concerning given the association with decreased survival and increased onward transmission. Even after informing our population-level 1^st^ and 2^nd^ 90’s by respondents’ VLS status, we still found that only a minority of HIV positive female sex workers were aware of their HIV status (45.5%), only 37.8% were on ART, and just 35.2% were virally suppressed. These estimates are much lower than UNAIDS population-level 90-90-90 targets of 90%, 81%, and 73%, respectively.

The HIV prevalence among female sex workers in Uganda is consistent with previously reported studies in 2009 [5, 20]. Sex work in Uganda remains criminalized; and enforcement of prohibitive sex-work policies are associated with a limited ability to negotiate safer sex practices and increased risk of HIV infection[21, 22]. Among the target population, 71.9% had been previously tested. Nevertheless, 28.1% of female sex workers indicated they had never been tested for HIV and 32.4% had not been tested in the last 12 months. These low estimates are concerning given that current recommendations suggest sex workers test every 6 to 12 months[17].

Among seropositive female sex workers, self-reported serostatus knowledge was low and likely to be an underestimate as demonstrated in previous studies[23]. We attempted to correct for this using viral load biomarker data. Under the assumption that female sex workers with VLS were aware of their HIV status and on ART, serostatus knowledge increased to 45.5%. Generally, serostatus knowledge among sex workers in sub-Saharan Africa has been notably low. In a 2011 Zimbabwe study, only half of HIV-positive female sex workers were aware of their status and less than 25% of negative female sex workers reported testing in the previous 6 months [24]. In three cities in Mozambique, serostatus knowledge among female sex workers ranged from 10.4 to 51.9% [25]. As with the general population, female sex workers face similar barriers to HIV testing including distance to the testing site, inconvenient schedules, and lack of awareness of services[26, 27]. In addition, fear of authorities, concerns about confidentiality, and that possibility that clients or others may learn their status and occupation, impede female sex workers from accessing services [28, 29]. Furthermore, female sex workers are more likely to experience gender-based violence as reported previously[30]. Almost half of female sex workers in our study population experienced rape in their lifetime and about one-third experienced three or more rape occurrences in the 6 months prior to the survey[30]. Increasing female sex workers empowerment has been associated with positive outcomes, including a reduction in HIV prevalence and increased utilization of health services[31, 32]. Developing more innovative approaches to delivering HIV testing and counseling services could increase female sex workersengagement.

There was marked discordance between actual serostatus and perceived serostatus. While 48.7% of seropositive women (among those previously tested for HIV) reported a previous positive test result, only 7.8% of seropositive women believed themselves to be positive and 79.3% indicated they did not know their status. We speculate that women on ART with VLS were more likely to believe they were no longer HIV-positive. Whether women are embarrassed or frightened to acknowledge their serostatus, or believe that ART is a ‘cure,’ additional education and counseling is urgently needed.

We speculate that the self-reported estimate for ART use is an underestimate. Female sex workers were only asked if they were on ART if they had previously reported a positive HIV test result. Like serostatus knowledge, we corrected our ART estimate by including women with VLS. Our estimate of 37.8% is similar to past studies in surrounding countries. In Mozambique, only 30–40% of eligible female sex workers self-reported ART use [25], and similarly in Zimbabwe, 26–38% of female sex workers self-reported ART use [24]. These estimates suggest the need to strengthen linkage to care among female sex workers.

VLS was low (35.2%) compared to other studies among female sex workers. In a 2014 Malawi study, almost half of HIV-positive female sex workers were virally suppressed [33]. Similar results were found in Zimbabwe, where 49.5% of female sex workers had viral loads of <1000 copies/ml [34]. We defined VLS as <1000 copies/ml, however other studies have used different thresholds, therefore we conducted a sensitivity analysis varying the definition of VLS[35, 36]. In Uganda, low service utilization as evidenced by the limited number of women on ART appears to be a major contributing factor in this population. The differences in time on ART and varied levels of adherence may also play a role[34]. For example, among female sex workers in Burkina Faso, high levels of viral suppression after 6 to 36 months on ART (79–82%) were correlated with high levels of ART adherence after 6 to 36 months (83–100%)[37]. Additionally, our corrected estimates could represent overestimates if the population includes a higher proportion of “elite controllers” who maintain undetectable viral loads without therapy, as evidenced in other studies conducted in Kenya and Uganda[38, 39].

There are number of limitations to our study. Female sex workers who received a coupon had to travel to the survey site to participate. Eligible female sex workers were then asked to provide self-reported data, which could be subject to recall bias. Social desirability bias could impact our results, as under-reporting of factors related to HIV, such as the number of clients, known serostatus, and self-reporting of ART are possible. We aimed to minimize these biases through the confidential nature of ACASI. Viral load data was not available for 29% of seropositive female sex workers and the questionnaire did not ask about the date of diagnosis, nor did it include time to treatment initiation or time since ART initiation, making qualification of VLS difficult. Additionally, HIV risk dynamics among female sex workers can vary substantially by the type and structure of the sex work and can change over time[40, 41]. Our survey questions included place of primary work, although it was not possible to select multiple locations and decipher changing environments over time. The illegality of sex work in Uganda likely influences the location of sex acts[42] and also may have discouraged participation in the survey. Moreover, RDS is dependent on the social connections of the recruitment participants and we could have missed women who were not socially connected. Additionally, only two of our seeds were productive. Our laboratory analyses were conducted in 2012, however guidance for HIV serological testing has since been updated[43]. Although our VL-adjusted estimates for serostatus knowledge and ART uptake represent a major strength in this analysis, it is possible that some women on ART may have failed to suppress viral load or may have been on ART not long enough to achieve VLS, and, therefore, may not have been classified as knowing their serostatus and being on ART.

Despite these limitations, the high prevalence of HIV and low proportion of HIV-positive female sex workers with VLS is concerning. Reduction of barriers to service utilization among both the general population and female sex workers must be addressed. Increasing testing uptake, more effective linkage to and uptake of ART, along with condom and pre-exposure prophylaxis (PrEP) provision by increasing service awareness and flexibility will be useful. Efforts that facilitate female sex workers’ ability to negotiate safer sex-work environments and criminalize abuse are urgently needed. Incorporation of community- and rights-based approaches with tailored prevention packages that included engagement of peer networks may reduce stigma and discrimination. Ongoing surveillance of HIV incidence and VLS in this population should occur to monitor trends in order to inform HIV control and prevention programs. A combination of intensified interventions promoting prevention and treatment are necessary if we are to achieve the ambitious 90–90–90 targets in Uganda.

## References

[1] UNAIDS. 90-90-90: an ambitious treatment target to help end the AIDS epidemic. UNAIDS, Geneva, Switzerland: 2014.

[2] Baral S, Beyrer C, Muessig K, Poteat T, Wirtz AL, Decker MR, et al Burden of HIV among female sex workers in low-income and middle-income countries: a systematic review and meta-analysis. Lancet Infect Dis. 2012;12(7):538–49. 10.1016/S1473-3099(12)70066-X . 22424777

[3] Prüss-Ustün A, Wolf J, Driscoll T, Degenhardt L, Neira M, Calleja JMG. HIV due to female sex work: regional and global estimates. PLoS One. 2013;8(5):e63476 10.1371/journal.pone.0063476 23717432 PMC3662690

[4] Uganda AIDS Commission and UNAIDS. Uganda HIV Modes of Transmission and Prevention Response Analysis. Kampala, Uganda: 2009.

[5] Hladik W, Baughman AL, Serwadda D, Tappero JW, Kwezi R, Nakato ND, et al Burden and characteristics of HIV infection among female sex workers in Kampala, Uganda–a respondent-driven sampling survey. BMC public health. 2017;17(1):565 10.1186/s12889-017-4428-z 28601086 PMC5466716

[6] Uganda Ministry of Health, ICF International. Uganda AIDS Indicator Survey, 2011 Calverton Maryland, USA: 2011.

[7] Beyrer C, Crago AL, Bekker LG, Butler J, Shannon K, Kerrigan D, et al An action agenda for HIV and sex workers. Lancet. 2015;385(9964):287–301. 10.1016/S0140-6736(14)60933-8 ; PubMed Central PMCID: PMCPMC4302059. 25059950 PMC4302059

[8] Deanna Kerrigan, Andrea Wirtz, Iris Semini, N'Della N'Jie, Anderson Stanciole, Butler Jenny, et al The Global HIV epidemics among Sex Workers. Washington, D.C.: World Bank; 2013.

[9] Penal Code Act 1950, (2000).

[10] Shannon K, Strathdee SA, Goldenberg SM, Duff P, Mwangi P, Rusakova M, et al Global epidemiology of HIV among female sex workers: influence of structural determinants. Lancet. 2015;385(9962):55–71. 10.1016/S0140-6736(14)60931-4 ; PubMed Central PMCID: PMCPMC4297548. 25059947 PMC4297548

[11] World Health Organization. Prevention and treatment of HIV and other sexually transmitted infections for sex workers in low and middle-income countries: Recommendations for a public health aaproach. Geneva: 2012. 26131541

[12] Staveteig Sarah, Wang Shanxiao, Head Sara K., Sarah E.K. Bradley, Nybro E. Demographic Patterns of HIV testing Uptake in Sub-Saharan Africa. Calverton, Maryland, USA: ICF Macro, 2013.

[13] Magnani R, Sabin K, Saidel T, Heckathorn D. Review of sampling hard-to-reach and hidden populations for HIV surveillance. AIDS. 2005;19 Suppl 2:S67–72. . 15930843 10.1097/01.aids.0000172879.20628.e1

[14] Heckathorn DD. Respondent-driven sampling: a new approach to the study of hidden populations. Social problems. 1997;44(2):174–99.

[15] Heckathorn DD. Respondent-driven sampling II: deriving valid population estimates from chain-referral samples of hidden populations. Social problems. 2002;49(1):11–34.

[16] Government of the Republic of Uganda: Ministry of Health. National Implementation Guidlines for HIV Counselling and Testing in Uganda. 2010.

[17] World Health Organization. Consolidated Guideliens on the Use of Antiretrovial Drugs for Treating and Preventing HIV Infection: Reommendations for a Public Health Approach. Geneva: 2016. 27466667

[18] Mark S. Handcock, Ian E. Fellows, Krista J. Gile. RDS Analyst: Software for the Analysis of Respondent-Driven Sampling Data. 2014.

[19] Gile KJ. Improved inference for respondent-driven sampling data with application to HIV prevalence estimation. Journal of the American Statistical Association. 2011;106(493):135–46.

[20] Vandepitte J, Bukenya J, Weiss HA, Nakubulwa S, Francis SC, Hughes P, et al HIV and other sexually transmitted infections in a cohort of women involved in high-risk sexual behavior in Kampala, Uganda. Sex Transm Dis. 2011;38(4):316–23. ; PubMed Central PMCID: PMCPMC3920055. 23330152 PMC3920055

[21] United Nations Development Programme. Global commission on HIV and the law: Risks, rights, and health. New York: UNDP, 2012.

[22] Shannon K, Strathdee SA, Shoveller J, Rusch M, Kerr T, Tyndall MW. Structural and environmental barriers to condom use negotiation with clients among female sex workers: implications for HIV-prevention strategies and policy. Am J Public Health. 2009;99(4):659–65. 10.2105/AJPH.2007.129858 ; PubMed Central PMCID: PMCPMC2661482. 19197086 PMC2661482

[23] Sanchez TH, Kelley CF, Rosenberg E, Luisi N, O'Hara B, Lambert R, et al Lack of Awareness of Human Immunodeficiency Virus (HIV) Infection: Problems and Solutions With Self-reported HIV Serostatus of Men Who Have Sex With Men. Open Forum Infect Dis. 2014;1(2):ofu084. 10.1093/ofid/ofu084 ; PubMed Central PMCID: PMCPMC4281805. 25734150 PMC4281805

[24] Cowan FM, Mtetwa S, Davey C, Fearon E, Dirawo J, Wong-Gruenwald R, et al Engagement with HIV prevention treatment and care among female sex workers in Zimbabwe: a respondent driven sampling survey. PLoS One. 2013;8(10):e77080 10.1371/journal.pone.0077080 ; PubMed Central PMCID: PMCPMC3797143. 24143203 PMC3797143

[25] Augusto Ado R, Young PW, Horth RZ, Inguane C, Sathane I, Ngale K, et al High Burden of HIV Infection and Risk Behaviors Among Female Sex Workers in Three Main Urban Areas of Mozambique. AIDS Behav. 2016;20(4):799–810. 10.1007/s10461-015-1140-9 ; PubMed Central PMCID: PMCPMC5092171. 26238035 PMC5092171

[26] Gage AJ, Ali D. Factors associated with self-reported HIV testing among men in Uganda. AIDS Care. 2005;17(2):153–65. 10.1080/09540120512331325635 . 15763711

[27] Maman S, Mbwambo J, Hogan NM, Kilonzo GP, Sweat M. Women's barriers to HIV-1 testing and disclosure: challenges for HIV-1 voluntary counselling and testing. AIDS Care. 2001;13(5):595–603. 10.1080/09540120120063223 . 11571006

[28] Mountain E, Pickles M, Mishra S, Vickerman P, Alary M, Boily MC. The HIV care cascade and antiretroviral therapy in female sex workers: implications for HIV prevention. Expert Rev Anti Infect Ther. 2014;12(10):1203–19. 10.1586/14787210.2014.948422 . 25174997

[29] Munoz J, Adedimeji A, Alawode O. 'They bring AIDS to us and say we give it to them': Socio-structural context of female sex workers' vulnerability to HIV infection in Ibadan, Nigeria. SAHARA J. 2010;7(2):52–61. . 21409295 10.1080/17290376.2010.9724957

[30] Schwitters A, Swaminathan M, Serwadda D, Muyonga M, Shiraishi RW, Benech I, et al Prevalence of rape and client-initiated gender-based violence among female sex workers: Kampala, Uganda, 2012. AIDS Behav. 2015;19 Suppl 1:S68–76. 10.1007/s10461-014-0957-y ; PubMed Central PMCID: PMCPMC4724433. 25432876 PMC4724433

[31] Kerrigan D, Moreno L, Rosario S, Gomez B, Jerez H, Barrington C, et al Environmental-structural interventions to reduce HIV/STI risk among female sex workers in the Dominican Republic. Am J Public Health. 2006;96(1):120–5. 10.2105/AJPH.2004.042200 ; PubMed Central PMCID: PMCPMC1470438. 16317215 PMC1470438

[32] Carlson CE, Chen J, Chang M, Batsukh A, Toivgoo A, Riedel M, et al Reducing intimate and paying partner violence against women who exchange sex in Mongolia: results from a randomized clinical trial. J Interpers Violence. 2012;27(10):1911–31. 10.1177/0886260511431439 ; PubMed Central PMCID: PMCPMC4269222. 22366477 PMC4269222

[33] Lancaster KE, Powers KA, Lungu T, Mmodzi P, Hosseinipour MC, Chadwick K, et al The HIV Care Continuum among Female Sex Workers: A Key Population in Lilongwe, Malawi. PLoS One. 2016;11(1):e0147662 10.1371/journal.pone.0147662 ; PubMed Central PMCID: PMCPMC4726447. 26808043 PMC4726447

[34] Cowan FM, Davey C, Fearon E, Mushati P, Dirawo J, Cambiano V, et al The HIV care cascade among female sex workers in Zimbabwe: results of a population-based survey from the Sisters Antiretroviral therapy Programme for Prevention of HIV, an Integrated Response (SAPPH-IRe) Trial. J Acquir Immune Defic Syndr. 2016 10.1097/QAI.0000000000001255 . 27930599

[35] Cook RL, Zhou Z, Kelso-Chichetto NE, Janelle J, Morano JP, Somboonwit C, et al Alcohol consumption patterns and HIV viral suppression among persons receiving HIV care in Florida: an observational study. Addict Sci Clin Pract. 2017;12(1):22 10.1186/s13722-017-0090-0 ; PubMed Central PMCID: PMCPMC5615807. 28950912 PMC5615807

[36] Ustinov A, Suvorova A, Belyakov A, Makhamatova A, Levina O, Krupitsky E, et al Psychiatric Distress, Drug Use, and HIV Viral Load Suppression in Russia. AIDS Behav. 2016;20(8):1603–8. 10.1007/s10461-016-1297-x ; PubMed Central PMCID: PMCPMC4945434. 26809193 PMC4945434

[37] Konate I, Traore L, Ouedraogo A, Sanon A, Diallo R, Ouedraogo JL, et al Linking HIV prevention and care for community interventions among high-risk women in Burkina Faso—the ARNS 1222 "Yerelon" cohort. J Acquir Immune Defic Syndr. 2011;57 Suppl 1:S50–4. 10.1097/QAI.0b013e3182207a3f . 21857287

[38] Kim AA, Mukui I, Young PW, Mirjahangir J, Mwanyumba S, Wamicwe J, et al Undisclosed HIV infection and antiretroviral therapy use in the Kenya AIDS indicator survey 2012: relevance to national targets for HIV diagnosis and treatment. AIDS. 2016;30(17):2685–95. 10.1097/QAD.0000000000001227 . 27782965 PMC6559732

[39] Jain V, Liegler T, Kabami J, Chamie G, Clark TD, Black D, et al Assessment of population-based HIV RNA levels in a rural east African setting using a fingerprick-based blood collection method. Clin Infect Dis. 2013;56(4):598–605. 10.1093/cid/cis881 ; PubMed Central PMCID: PMCPMC3552523. 23243180 PMC3552523

[40] Bekker LG, Johnson L, Cowan F, Overs C, Besada D, Hillier S, et al Combination HIV prevention for female sex workers: what is the evidence? Lancet. 2015;385(9962):72–87. 10.1016/S0140-6736(14)60974-0 . 25059942 PMC10318470

[41] Harcourt C, Donovan B. The many faces of sex work. Sex Transm Infect. 2005;81(3):201–6. 10.1136/sti.2004.012468 ; PubMed Central PMCID: PMCPMC1744977. 15923285 PMC1744977

[42] World Health Organization. Violence against sex workers and HIV prevention. Geneva: World Health Organization, 2005.

[43] UNAIDS. Monitoring HIV Impact Using Population-Based Surveys. Geneva, Switzerland: 2015.

